# Supplementary material for: Lysine Inhibits Hemolytic Activity of Staphylococcus aureus and Its Application in Food Model Contaminated with Staphylococcus aureus
Source: Toxins (Basel). 2022 Dec 9;14(12):867. doi: 10.3390/toxins14120867 (PMC9786064; doi:10.3390/toxins14120867)
Supplement: Supplementary file 1 [file toxins-14-00867-s001.zip › toxins-2049562-supplementary.pdf]

Table S1. Lamb Sensory Scoring Standard.

Sensory Score	Color	Organization Status	Odor	Overall Acceptability
9–10	bright, shiny	dense flesh, flexible	normal fresh meat taste	like very much
8–9	slightly dim, slightly dull	denser flesh, good elasticity	normal meat taste, weak fishy smell	like
6–8	darker, less glossy	slightly soft, slightly less elastic	Slight fishy and peculiar smell	generally
3–5	dim, matt	soft, less elastic	Obvious fishy smell and odor	slightly dislike
0–2	very dull, matt	very soft flesh, very poor elasticity	strong fishy odor and odor	dislike
